# Association of suboptimal health status with intestinal microbiota in Chinese youths

**DOI:** 10.1111/jcmm.14880

**Published:** 2019-12-06

**Authors:** Qi Sun, Xizhu Xu, Jie Zhang, Ming Sun, Qiuyue Tian, Qihuan Li, Weijie Cao, Xiaoyu Zhang, Hao Wang, Jiaonan Liu, Jinxia Zhang, Xiaoni Meng, Lijuan Wu, Manshu Song, Hongqi Liu, Wei Wang, Youxin Wang

**Affiliations:** ^1^ Beijing Key Laboratory of Clinical Epidemiology School of Public Health Capital Medical University Beijing China; ^2^ National Research Institute for Family Planning Beijing China; ^3^ Graduate School of Peking Union Medical College Beijing China; ^4^ School of Public Health Shandong First Medical University & Shandong Academy of Medical Sciences Taian China; ^5^ University Hospital Weifang University Weifang China; ^6^ School of Medical and Health Sciences Edith Cowan University Perth WA Australia

**Keywords:** 16S rRNA, intestinal microbiota, LEfSe analysis, random forest tree, suboptimal health status

## Abstract

Suboptimal health status (SHS), a physical state between health and disease, is a subclinical and reversible stage of chronic disease. Previous studies have shown alterations in the intestinal microbiota in patients with some chronic diseases. This study aimed to investigate the association between SHS and intestinal microbiota in a case‐control study with 50 SHS individuals and 50 matched healthy controls. Intestinal microbiota was analysed by MiSeq 250PE. Alpha diversity of intestinal microbiota in SHS individuals was higher compared with that of healthy controls (Simpson index, *W* = 2238, *P* = .048). Beta diversity was different between SHS and healthy controls (*P* = .018). At the phylum level, the relative abundance of Verrucomicrobia was higher in the SHS group than that in the controls (*W* = 2201, *P* = .049). Compared with that of the control group, nine genera were significantly higher and five genera were lower in abundance in the SHS group (all *P* < .05). The intestinal microbiota, analysed by a random forest model, was able to distinguish individuals with SHS from the controls, with an area under the curve of 0.79 (95% confidence interval: 0.77‐0.81). We demonstrated that the alteration of intestinal microbiota occurs with SHS, an early stage of disease, which might shed light on the importance of intestinal microbiota in the primary prevention of noncommunicable chronic diseases.

## 1 INTRODUCTION

Suboptimal health status (SHS) is a physical state between health and disease and is characterized by the symptoms of health complaints, general weakness and low energy within a period of 3 months.^1^ A comprehensive Suboptimal Health Status Questionnaire‐25 (SHSQ‐25) was used to assess SHS, with the SHSQ‐25 accounting for the multidimensionality of SHS by assessing the following: (a) fatigue, (b) the cardiovascular system, (c) the digestive tract, (d) the immune system and (e) mental status.^2^ To date, the SHSQ‐25 has been validated in various populations, including Chinese,^3^ African^4^ and European populations.^5^ Although several items of SHS are similar to some diseases, SHS cannot reach the diagnosable condition of any current defined diseases. SHS is not a disease state, but several studies have suggested that SHS, as an overall assessment of human body, might precede the occurrence of noncommunicable chronic diseases (NCDs), including type 2 diabetes mellitus (T2DM), cardiovascular diseases and hypertension.^3^, ^4^, ^5^, ^6^ A cross‐sectional study conducted among workers in urban Beijing demonstrated that SHS was associated with cardiovascular risk factors.^6^ In Russia, a community‐based cross‐sectional study showed that SHS was related to endothelial dysfunction, suggesting that the integration of SHS and endothelial dysfunction can be applied to the elevated risk of cardiovascular diseases.^5^ In Ghana, a cross‐sectional study demonstrated that SHS might be involved in the development of T2DM.^4^ In a cross‐sectional study of Chinese students, SHS was shown to be correlated with stress management, psychological states and physical activity.^7^ More recently, Anto et al^8^ found that high SHS score is associated with increased incidence of preeclampsia.

In China, due to modern lifestyles, increased work pressures, changes in diet and other factors, the prevalence of chronic diseases such as hypertension, heart disease, stroke, cancer, chronic obstructive pulmonary disease and diabetes is increasing*.* 9 As a subclinical and reversible stage of chronic disease, SHS might play a very important role in the pathogenesis of chronic diseases.^2^ The aetiology and mechanism of SHS are still poorly understood, and SHS is diagnosed based on subjective questionnaires and lacks objective biomarkers. Shortened relative telomere length, *N*‐glycosylation and high levels of plasma cortisol were associated with SHS, suggesting that objective biomarkers have the potential to diagnose SHS.^10^, ^11^, ^12^ Therefore, novel diagnostic markers based on objective measurements are urgently needed.

In recent years, many studies have shown that intestinal microbiota play important roles in host health,^13^ as well as the immune system,^14^, ^15^ nervous system,^16^ digestive system^17^ and cardiovascular system,^18^ all of which are components of SHS.^2^ Emerging evidence suggests a link between the intestinal microbiota and various diseases, such as atherosclerosis,^19^, ^20^ hypertension,^21^ T2DM^22^ and acquired immune deficiency syndrome.^23^ This association between microbiota and diseases was also validated through faecal transfer experiments.^24^, ^25^, ^26^ Furthermore, altered gut microbiota was observed in patients suffering from chronic fatigue syndrome (CFS), a disease resembling SHS.^27^, ^28^ Fremont et al^27^ reported that intestinal microbiota is altered in myalgic encephalomyelitis/CFS patients from Belgium and Norway. Giloteaux et al^28^ found dysbiosis and translocation of the intestinal microbiota in CFS. Because of the association between intestinal microbiota and NCDs, together with SHS as an early stage of NCDs, we hypothesized that the alternation of intestinal microbiota might occur in SHS patients and that the interaction might increase the incidence of NCDs. In this study, we attempted to profile microbiota composition to identify novel bacteria associated with SHS.

## 2 MATERIALS AND METHODS

### 2.1 Study population and design

Previously, we conducted a cross‐sectional survey among undergraduates at Weifang University, which is located in Shandong Province, China, in November 2017. In total, 5219 participants completed the questionnaire, of which 442 (8.50%) were considered suffering from SHS.^29^ Among these undergraduates, 50 cases and 50 controls were included in this case‐control study. All students attended a standardized examination protocol in Weifang Hospital, including an interview about the history of previous diseases, family income, physical activity, smoking status and history of drinking. They received a blood biochemical examination and were asked to complete the SHSQ‐25. SHSQ‐25 includes 25 items: (a) item 1 to 6 and item 8 to 10 are used to measure symptoms of fatigue; (b) item 18 to 24 are used to measure symptoms of mental status; (c) item 11 to 13 are used to measure symptoms of the cardiovascular system; (d) item 7, 17 and 25 are used to measure symptoms of immune system; (e) item 14 to 16 are used to measure symptoms of the digestive tract (Table S1).^2^ Participants were enrolled if they met the following inclusion criteria: (a) signed informed consent; and (b) a completed questionnaire. They were excluded if they met the following exclusion criteria: (a) a history of somatic or psychiatric abnormalities; (b) a history of mental illness or drug abuse; and (c) a history of antibiotic consumption in the previous 2 months. After enrolment, propensity score matching was used to match a subset of 50 SHS cases (SHS score ≥35) to a subset of 50 ideal healthy controls (SHS score <35) by age, gender and body mass index (BMI). The score was calculated from the SHSQ‐25. The details of SHSQ‐25 and the characteristics of the subjects between SHS group and control group are shown in Tables 1 and S2.

**Table 1:** Characteristics of subjects in SHS and healthy groups

Variables	Healthy group# (n = 50)	SHS group# (n = 50)	*χ* ^2^/*t*	*P*
Age (y)	18.96 ± 0.64	19.00 ± 0.73	−0.29^a^	.77
Gender, male (%)	11 (22)	10 (20)	0.06^b^	.81
BMI (kg/m^2^)	22.03 ± 2.72	21.67 ± 3.03	0.62^a^	.53
WHR	0.77 ± 0.05	0.77 ± 0.05	0.41^a^	.68
Sleeping time (h)	7.21 ± 1.20	6.92 ± 1.48	−1.06^a^	.29
Physical activity, n (%)			3.58^b^	.17
Inactive	1 (2)	4 (8)		
Moderately	15 (30)	20 (40)		
Very active	34 (68)	26 (52)		
Family income, n (%)			0.56^b^	.75
≤¥3000	22 (44)	21 (42)		
¥3001‐5000	19 (38)	17 (34)		
≥¥5001	9 (18)	12 (24)		
No smoking, n (%)	50 (100)	49 (98)	1.01^b^	.31
No drinking, n (%)	50 (100)	50 (100)	–	–
SBP (mm Hg)	120.90 ± 11.51	118.30 ± 12.54	1.06^a^	.29
DBP (mm Hg)	77.18 ± 8.06	75.71 ± 10.80	0.77^a^	.45
ALT (μg/L)	15.58 ± 9.14	15.48 ± 8.54	0.06^a^	.96
AST (μg/L)	21.84 ± 5.63	22.38 ± 5.91	−0.46^a^	.65
CKMB (μg/L)	9.26 ± 2.63	7.18 ± 4.46	0.11^a^	.91
ALP (μg/L)	62.17 ± 15.27	62.76 ± 18.20	−0.17^a^	.86
TBIL (μg/L)	15.60 ± 6.69	14.07 ± 5.89	1.19^a^	.24
LDH (μg/L)	147.40 ± 23.08	142.70 ± 21.96	1.02^a^	.31
Total score of SHSQ‐25	17.58 ± 8.09	42.88 ± 7.24	−16.48^a^	<.0001
Fatigue score	7.16 ± 3.48	16.70 ± 2.88	−14.92^a^	<.0001
Cardiovascular system score	0.44 ± 0.88	3.42 ± 2.15	−9.07^a^	<.0001
Digestive tract score	1.72 ± 1.55	4.50 ± 1.99	−7.78^a^	<.0001
Immune system score	2.68 ± 1.43	5.08 ± 1.60	−7.89^a^	<.0001
Mental status score	5.58 ± 3.41	13.18 ± 4.20	−9.94^a^	<.0001

Abbreviations: ¥, Chinese Yuan; ALP, alkaline phosphatase; ALT, alanine aminotransferase; AST, aspartate aminotransferase; BMI, body mass index; CKMB, creatine kinase isoenzymes; DBP, diastolic blood pressure; LDH, lactate dehydrogenase; SBP, systolic blood pressure; SHS, suboptimal health status; SHSQ‐25, Suboptimal Health Status Questionnaire‐25; TBIL, total bilirubin; WHR, waist‐to‐hip ratio.

^#^ Continuous variables were expressed as mean ± standard deviation, while discrete variables were represented as number (proportion); a, *t* value; b, *χ* ^2^ value.

### 2.2 Faecal sample collection and DNA extraction

Stool samples were obtained through a specimen collection kit and immediately stored at −80°C. Bacterial DNA was extracted at the Beijing Municipal Key Laboratory of Clinical Epidemiology using a TIANGEN kit based on the manufacturer's recommendations. The concentration and purity were evaluated using a Nanodrop^®^ spectrophotometer (Thermo Scientific). Extracted DNA was stored at −80°C.

### 2.3 DNA sequencing of the 16S rRNA gene

The V4 region of the bacterial 16S rRNA gene was amplified from extracted faecal DNA through degenerate primers (515F/806R: forward GTGCCAGCMGCCGCGGTAA, reverse GGACTACHVGGGTWTCTAAT). The amplicons were purified and quantified, according to manufacturer's protocols by Beijing Cheerland Biotech Co., Ltd, China. The Polymerase chain reaction (PCR) amplification was carried out in a 20 μL reaction system containing 4 μL 5× FastPfu Buffer, 2 μL 2.5 mmol L^−1^ dNTPs, 0.8 μL Forward Primer (5 μmol L^−1^), 0.8 μL Reverse Primer (5 μmol L^−1^), 0.4 μL FastPfu Polymerase DNA, 10 ng Template DNA and supplementary distillation‐distillation H_2_O (Cheerland Biotech). The procedure of PCR is as follows: denaturation (30 seconds at 98°C), followed by 27 cycles consisting of denaturation (15 seconds at 98°C), annealing (15 seconds at 58°C), extension (15 seconds at 72°C) and a final extension at 72°C for 1 minutes. PCR products were detected by 1% agarose gel electrophoresis. The magnetic bead system was used to purify the replicate PCR reactions. Purified PCR amplicons of each sample were mixed, according to the amplicon concentration of samples detected by Nanodrop. The amplicons were sequenced in a single pool in one run with the MiSeq platform (250 PE, Illumina), generating approximately 5.76 Gb of data. After the raw sequences were processed to concatenate forward and reverse reads, paired‐end sequences and barcodes were sorted and matched.

### 2.4 Bioinformatic analysis

Quality filtering and analyses of microbial community diversity were performed with the QIIME1 software package (Quantitative Insights into Microbial Ecology). Sequences were removed from the analysis if they were <200 nt, had a quality score <20, contained ambiguous characters, contained an uncorrectable barcode or did not contain the primer sequence. Sequences were assigned to samples by examining their individual 12‐nt barcode. Sequences were aligned through PyNAST and clustered into OTUs by 97% similarity, while taxonomy was assigned using the Ribosomal Database Project as a reference base. The phylogenetic tree was built with FastTree. QIIME1 and UniFrac were used for analyses of bacterial communities and group comparisons. Alpha diversity (Chao1 index, Shannon index, Simpson index and phylogenetic diversity index) was calculated in QIIME1. Across all specimens, the beta diversity distance metrics, including unweighted and weighted UniFrac distance metrics, were calculated and visualized by principal coordinates analysis (PCoA). To find features differentially represented between healthy and SHS groups, linear discriminant effect size analysis (LEfSe)^30^ based on OTU level was performed. LEfSe, an algorithm for high‐dimensional biomarker discovery, used linear discriminant analysis (LDA) to estimate the effect size of each taxon differentially represented in SHS and controls.

### 2.5 Statistical analysis

The Kolmogorov‐Smirnov test was used for normality test. If the data conformed to a normal distribution, continuous variables were presented as the mean ± standard deviation (SD), and an independent *t* test was used for analysis. If data were not normally distributed, continuous variables were presented as a median (interquartile range) and analysed by Wilcoxon rank‐sum test. The χ^2^ test was used to examine differences in categorical variables between the cases and controls. Wilcoxon rank‐sum tests were used to assess associations of individual taxon relative abundances. Alpha diversity was compared across groups by Wilcoxon rank‐sum tests. In order to evaluate the discriminative capability of intestinal microbiota, a random forest model was established using 10‐fold cross‐validation, and the performance of the random forest tree model was measured as the area under the curve (AUC). We firstly selected variables using stepwise method in the logistic regression and then selected variables which are statistically differed in the SHS and controls. Finally, these variables were entered into random forest model under full factory design, and the model with highest AUC value was selected as the best model.

All results were deemed significant if the *P*‐value was below .05. The data analysis was performed using IBM SPSS Statistics software (version 20.0 from Armonk, NY: IBM Corp) and R software version 3.4.2.

### 2.6 Ethics statement

This study was approved by the ethics committee of Capital Medical University, Beijing, China. All subjects gave written informed consent in accordance with the Declaration of Helsinki.

## 3 RESULTS

### 3.1 Demographic profiles of SHS and the controls

In total, 50 SHS individuals consisting of 40 female and 10 male participants with an average age of 19.00 ± 0.73 years, and 50 healthy students with similar demographic features, composed of 39 female and 11 male individuals with an average age of 18.96 ± 0.64 years, were selected in the final analysis. The SHSQ‐25 score of the SHS and control group was 42.88 ± 7.24 and 17.58 ± 8.09, respectively (*P* < .0001). Five dimensions of SHS were significantly different between the healthy and SHS groups (cardiovascular system: 0.44 ± 0.88 vs 3.42 ± 2.15, fatigue: 7.16 ± 3.48 vs 16.7 ± 2.88, digestive tract: 1.72 ± 1.55 vs 4.5 ± 1.99, immune system: 2.68 ± 1.43 vs 5.08 ± 1.6, mental status: 5.58 ± 3.41 vs 13.18 ± 4.2; all *P* < .0001, Table 1). A cluster analysis was used to describe SHSQ‐25 for all individuals. The SHS and healthy samples can be clustered separately in different parts, and the cluster tree was shown in Figure S1. There were no significant differences between the SHS and control groups in demographic variables (age, sex, family income, alcohol consumption, smoking history and physical activity), anthropometric measurements (BMI and WHR), blood pressure or biochemical measurements (Table 1).

### 3.2 Microbial diversity

**Figure 1:**
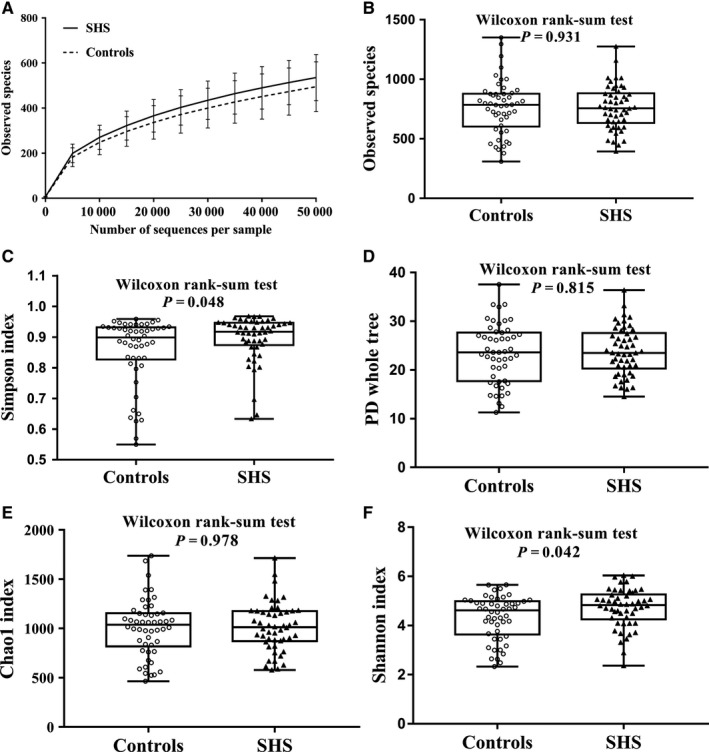
Rarefaction curves and comparison of alpha diversity between the gut microbiota of SHS and controls (50 cases & 50 controls). Rarefaction curves for the observed species of healthy individuals and SHS individuals (A). Five indices were used to represent the alpha diversity, which is Observed species (B), Simpson index (C), PD whole tree (D), Chao1 index (E) and Shannon index (F). PD indicates phylogenetic diversity

A total of 16 860 320 16S rRNA reads were obtained from the 100 samples, with an average of 168 603 ± 97 006 reads per sample. After the quality filtering, 15 778 975 16S rRNA reads remained, with an average of 157 790 ± 85 638 reads per sample. The number of sequences in the smaller sample is 29 154 reads which was used to compensate for differential sequencing depth. Sequences were classified into 764 ± 204 operational taxonomic units (OTUs) per sample at the identity threshold of 97%, and 1.64% reads were not clustered as OTU (Figure S2). The sequence‐based rarefaction curves based on the observed species were nearly asymptotic (Figure 1A). SHS individuals had higher microbial diversity compared with that of healthy individuals (Simpson index: Median 0.92 (P_25_‐P_75_: 0.87‐0.94) vs 0.90 (0.83‐0.93), *P* = .048; Shannon: 4.83 (4.25‐5.26) vs 4.61 (3.64‐4.99), *P* = .042). There is no difference in microbial richness indices between SHS individuals and controls (observed species: median 757.00 (P_25_‐P_75_: 632.50‐882.50) vs 785.50 (602.5‐878.75), *P* = .931; phylogenetic diversity: 23.50 (20.27‐27.59) vs 23.61 (17.71‐27.68), *P* = .815; Chao1: 1011.89 (871.22‐1176.25) vs 1037.91 (817.12‐1153.75), *P* = .978; Figure 1, Table S3).

**Figure 2:**
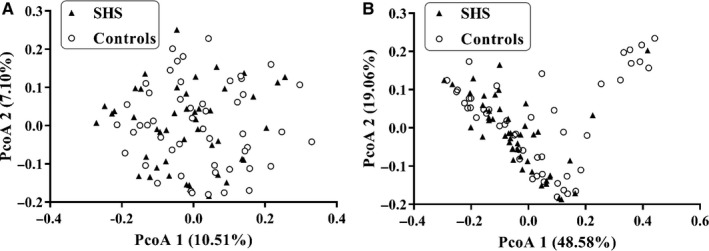
Principal coordinate analysis illustrating the grouping patterns of the SHS and control individuals based on the unweighted UniFrac (A) (*P* = .145) and weighted UniFrac (B) distances (50 cases & 50 controls, *P* = .018)

Weighted and unweighted UniFrac distance metric matrices were used to evaluate overall differences between the two groups through PCoA. Weighted UniFrac distance metrics in some extent distinguished the intestinal microbiota of SHS subjects from that of the control subjects (*P* = .018). There appeared to be a difference in beta diversity between the SHS group and healthy group (Figure 2). On the weighted UniFrac distance metrics figure, there is a cluster of nine healthy and one SHS at the top right corner that is clearly separated from the rest of the individuals. The aspartate aminotransferase (AST) and alanine aminotransferase (ALT) were found to be increased in the 10 individuals (Table S4). In order to find the relationship between ALT/AST and intestinal microbiota, a Spearman's correlation analysis was performed. In phylum level, ALT had a weak positive correlation with Euryarchaeota (*r* = .28, *P* = .01), and AST had weak positive correlation with Actinobacteria (*r* = .26, *P* = .01) and Synergistetes (*r* = .23, *P* = .03; Table S5).

### 3.3 Composition of faecal bacteria

**Figure 3:**
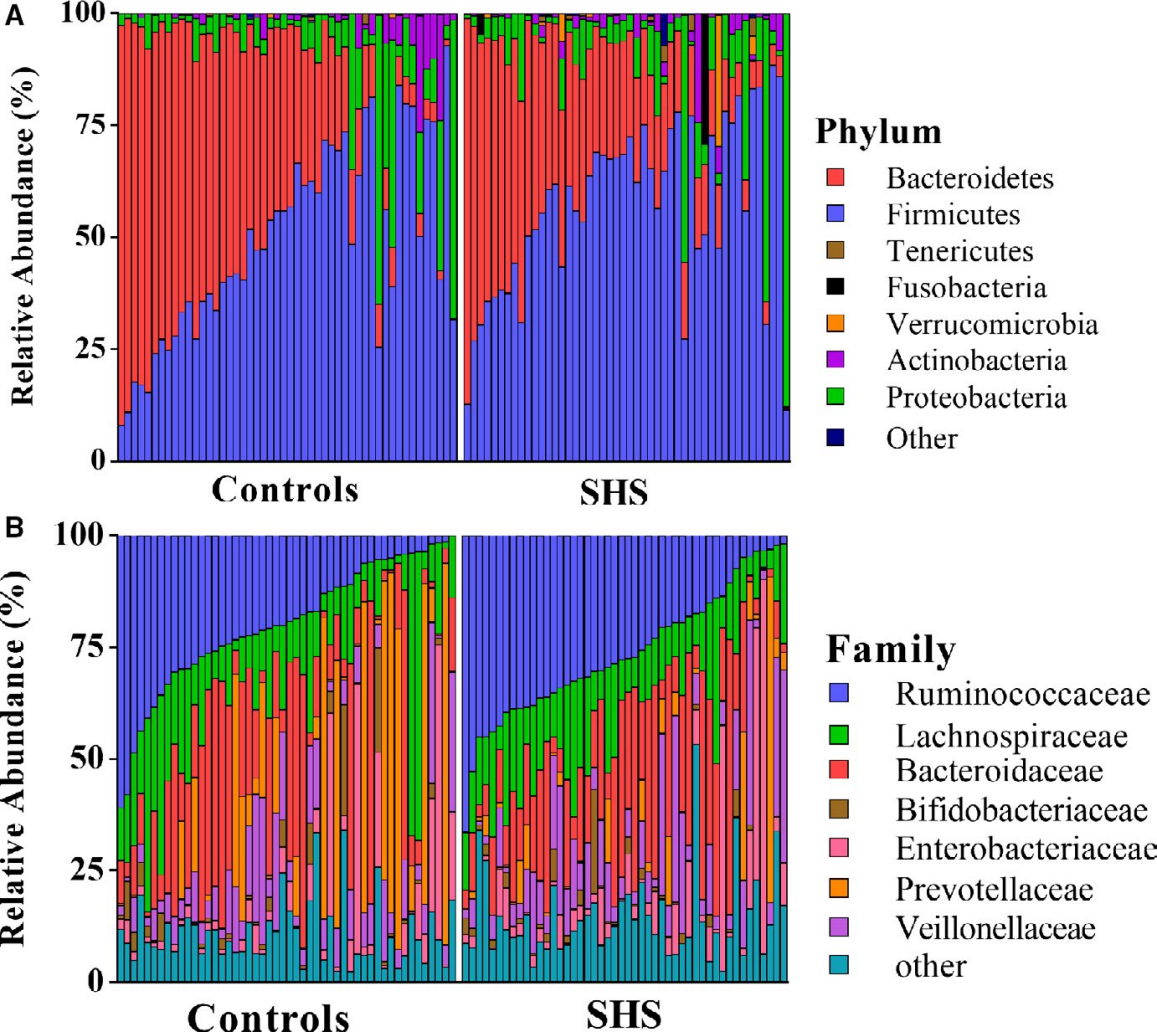
Composition of the intestinal microbiota of healthy individuals and SHS patients in phylum level (A) and family‐level (B) gut microbial taxa (50 cases & 50 controls)

At the phylum level, the majority of the 16S reads were classified into four phyla: Firmicutes (52.92%), Bacteroidetes (33.41%), Proteobacteria (9.99%) and Actinobacteria (2.44%). The two largest phyla represented in each dataset of controls and SHS groups were Firmicutes and Bacteroidetes (Figure 3). Verrucomicrobia, Fusobacteria, Tenericutes, Euryarchaeota, TM7, Cyanobacteria and Synergistetes were also detected, representing 1% of the total reads analysed. At the phylum level, the relative abundance of Verrucomicrobia was considered to be higher in the SHS group than in the controls (*P* = .049; Table S6). There was no significant difference in ratio of Firmicutes/Bacteroidetes between SHS and control group (*P* = .11, Table S6).

**Figure 4:**
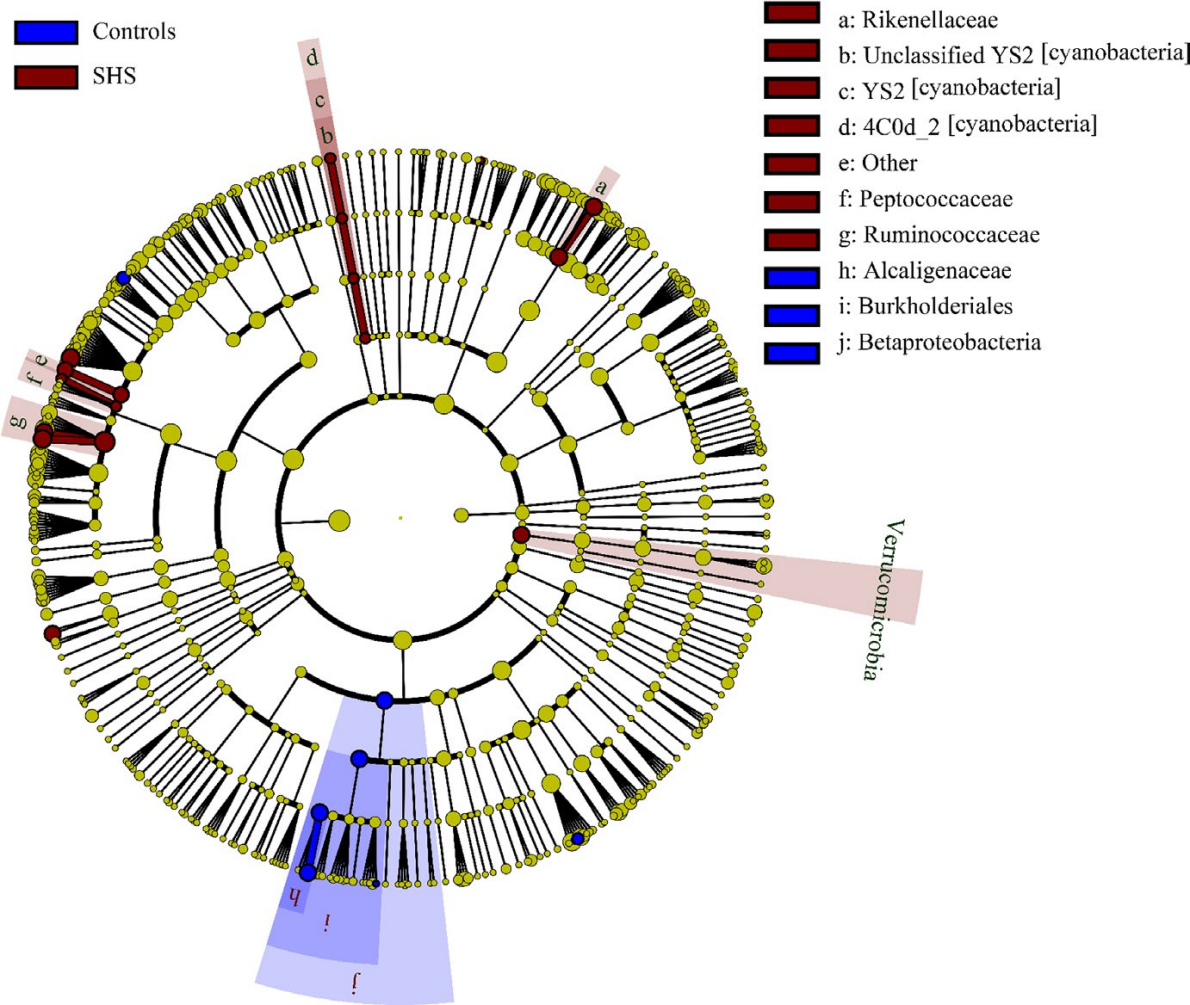
Phylogenetic dendrogram of biomarker bacteria between SHS and control groups (50 cases & 50 controls)

**Figure 5:**
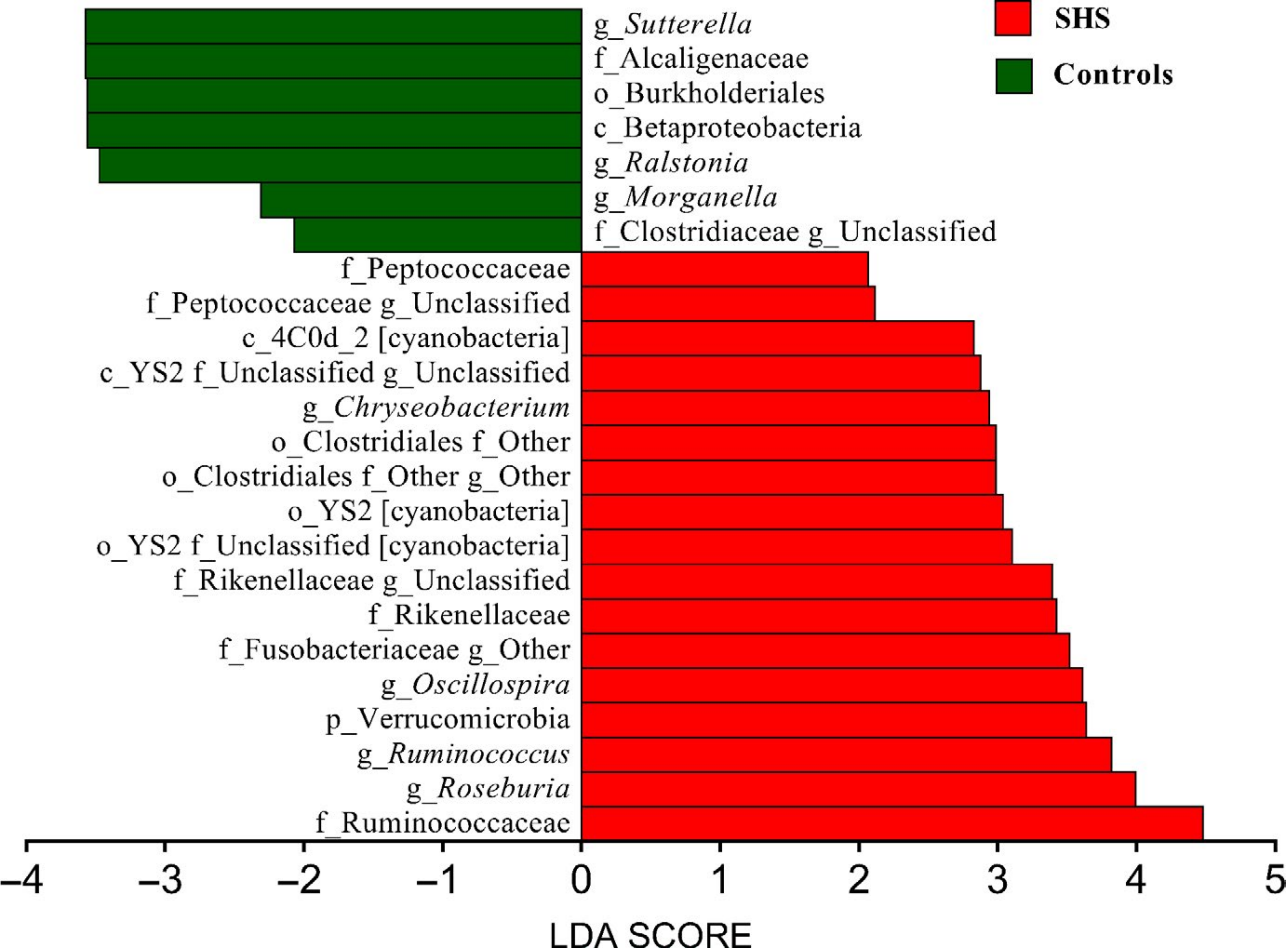
Linear Discriminant Analysis (LDA) score of LEfSe analysis between SHS and control groups (50 cases & 50 controls, LDA score ≥2)

At the genus level, 352 different bacterial genera were identified from SHS and controls in the study. The genera represented in almost all subjects were *Bacteroides* (19.52%), *Faecalibacterium* (15.25), *Prevotella* (10.21%), *Escherichia* (5.52%), *Dialister* (4.82%), *Roseburia* (4.03%) and *Ruminococcus* (3.5%). In SHS group, the comparative abundances of *Oscillospira, Pseudobutyrivibrio, Roseburia, Ruminococcus, Chryseobacterium,* unclassified Clostridiaceae, unclassified Rikenellaceae, unclassified Fusobacteriaceae and unclassified Peptococcaceae were higher, while the relative abundances of Clostridiaceae *02d06*, *Sutterella*, *Ralstonia*, *Morganella* and unclassified Peptococcaceae were lower compared with those of the healthy group. (*P* < .05, Table S6; Figure S3). LEfSe analysis, an algorithm for high‐dimensional biomarker discovery, further confirms these significant differences. Twenty‐four discriminative features (LDA score ≥2) with relative abundances varied significantly between the SHS group and the control group (Figures 4 and 5). Taxon annotation of OTUs from all samples was shown in Table S7.

### 3.4 Using a random forest model to discriminate between the SHS group and the control group

**Figure 6:**
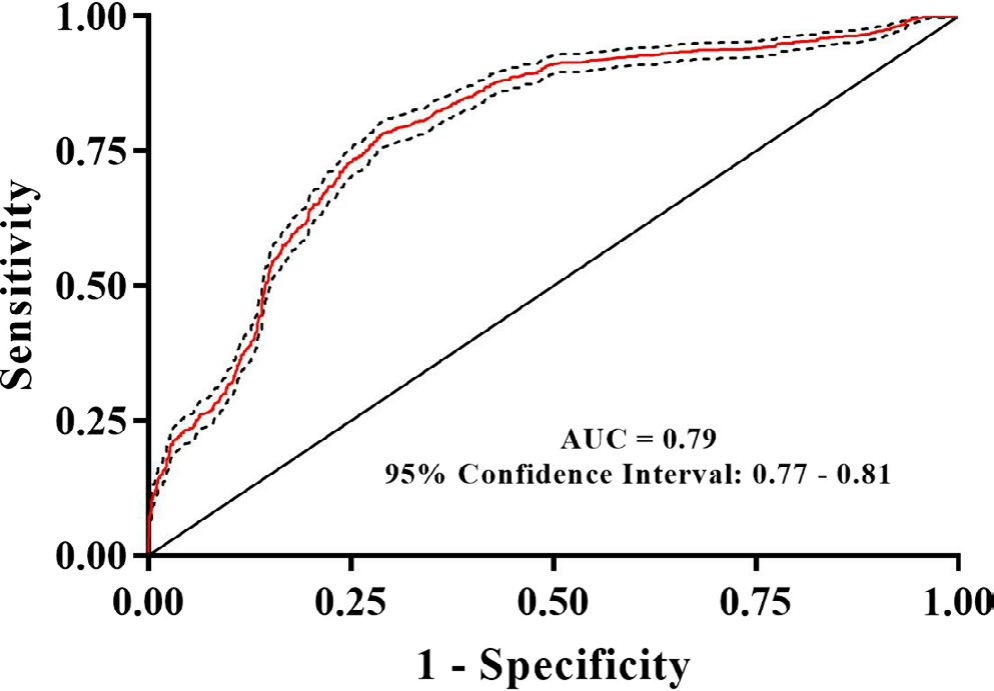
Receiver operating characteristic (ROC) curve analysis of random forest analysis in the diagnosis of SHS

A random forest approach was used to classify samples into SHS or control groups. The random forest diagnosis model was validated by 10‐fold cross‐validation. The AUC for the random forest diagnostic model of SHS was 0.79 (95% CI: 0.77‐0.81; Figure 6). The confusion matrix of the model is shown in Table 2.

**Table 2:** Confusion matrix for random forest analysis

Source	Predicted	
	Controls	SHS
Controls	37.58	13.44
SHS	12.60	36.38

Note

Values are presented as percentage.

Abbreviation: SHS, suboptimal health status.

## 4 DISCUSSION

In the present study, alpha diversity of intestinal microbiota in SHS individuals was significantly higher compared with that of healthy controls. In addition, beta diversity was significantly different between SHS and healthy controls, but the difference is not big enough. Sixteen intestinal bacterial biomarkers of SHS were identified by LEfSe analysis. The intestinal microbiota can effectively discriminate individuals with SHS from the controls, with an AUC of 0.79 (95% CI: 0.77‐0.81). This is the first attempt to explore the association between SHS and intestinal microbiota.

Alpha diversity, the mean species diversity of habitats at a local scale, includes two levels: richness and evenness.^31^ Kostic et al^32^ reported that microbial richness and the diversity of individuals decreased before the onset of type 1 diabetes. Li et al^21^ and Yan et al^33^ showed that the microbial richness and diversity of patients with hypertension were dramatically decreased. In the present study, there is no difference of the microbial richness (Chao1 index, observed species and phylogenetic diversity) between SHS individuals and healthy controls. However, the Simpson index and Shannon index, a comprehensive indicator of richness and evenness, in SHS individuals were higher than that of controls, suggesting that microbial diversity of SHS is higher than that of controls. The phenomenon of microbial diversity decreasing in disease status but increasing in SHS remains a mystery. The strict exclusion criteria in the definition of SHS and ideal health status, together with the homogeneity of youth undergraduates, might partly explain these disparities. Additionally, we showed that the beta diversity, determined by weighted UniFrac distance metrics, can significantly discriminate SHS subjects from control subjects, suggesting that changed diversity of intestinal microbiota occurs in SHS before the onset of NCDs. In the Figure 2B, 10 individuals (nine controls and one SHS) were clearly separated from other individuals. The AST and ALT of these 10 individuals were within normal range, but significantly higher than those of remains, might suggesting that there is an association between intestinal microbiota and transaminases. In phylum level, ALT had a weak positive correlation with Euryarchaeota, and AST had weak positive correlation with Actinobacteria and Synergistetes. Euryarchaeota, belong to archaea, is mainly composed of methanogenic bacteria which indirectly affects the health of the human body.^34^ Abundance of Actinobacteria increased in acute‐on‐chronic alcohol mice while decreased in cirrhotic patients.^35^, ^36^ Increased Synergistetes was found in bile from individuals with opisthorchiasis.^37^ As mentioned above, these bacterial abundances are altered in several diseases, but it is difficult to clarify how these bacteria affect the level of ALT/AST.

Alterations of intestinal microbiota composition are suspected to affect the host inflammatory and metabolic responses, and several studies have shown that disruption of intestinal microbiota equilibrium was associated with chronic diseases.^38^, ^39^, ^40^, ^41^ In our study, the large majority of all sequences identified belonged to one of four phyla, Firmicutes, Bacteroidetes, Proteobacteria or Actinobacteria, consistent with previous reports from human intestinal microbiota studies.^42^ However, the relative proportions of Verrucomicrobia were significantly higher in SHS individuals than in healthy controls. Previous study found that the relative proportions of Verrucomicrobia increased in the intestine following broad‐spectrum antibiotic treatment.^43^ This difference may be attributed to a medication history of antibiotic use. Participants with SHS are susceptible to diseases, and they have poor resistance to disease. Therefore, the frequency of antibiotic consumption might be higher than those of healthy. Although participants have a history of antibiotic consumption in the previous 2 months were excluded, participates used antibiotic before 2 months might affect the intestinal microbiota. In another study, the abundance of Verrucomicrobia was found to be modulated by the immune system,^44^ and the immune system is a component of SHS, suggesting SHS leads to inflammation which causes changes in intestinal microbiota. In our study, five genera, including *Oscillospira*, *Pseudobutyrivibrio*, *Roseburia*, *Ruminococcus* and *Chryseobacterium,* were increased compared with those in controls. Konikoff *et al* reported that *Oscillospira* is associated with lower BMI and inflammatory diseases and *Oscillospira* may utilize mammalian‐derived glycans from host or animal protein‐rich diet.^45^ Some *Oscillospira* species can probably produce the important short‐chain fatty acid butyrate which is important for prevention of inflammation.^46^, ^47^ A recent study showed that the relative abundance of *Oscillospira* is increased in patients with gallstones compared with that of controls.^48^ The mechanism of cholesterol gallstone disease is associated with inflammation,^49^ suggesting that changes in *Oscillospira* may be associated with inflammation. *Ruminococcus*, a member of the family *Ruminococcaceae*, has been reported to increase in colorectal cancer,^50^ Alzheimer's disease^51^ and type 1 diabetes,^52^ but decreased in Crohn's disease.^53^, ^54^ *Ruminococcus* is a butyrate‐producing bacterium, and butyrate has a positive effect on human health through anti‐inflammatory effects. In this study, we observed that *Ruminococcus* decrease in SHS, and this inconsistence remains unexplainable. Increased *Pseudobutyrivibrio* and *Roseburia* have been observed in patients with acute diverticulitis^55^ and Hashimoto's thyroiditis,^56^ respectively. *Chryseobacterium,* a gram‐negative bacterium, causes endogenous infection due to low host immunity and the irrational use of antibiotics.^57^ In addition, SHS individuals were also characterized by a significant reduction in Clostridiaceae *02d06*, *Sutterella*, *Ralstonia* and *Morganella*. Clostridiaceae *02d06* was enriched in T2DM patients compared with that in individuals without diabetes.^58^ Decreased abundance of *Sutterella* and *Ralstonia* were observed in patients with ulcerative colitis (UC)^40^ and atherosclerosis,^59^ respectively. *Sutterella* is considered to be a microorganism that induces a protective immune regulation profile.^60^ The above discussion suggests that alterations of intestinal microbiota in the SHS may involve diet and inflammation. In the LEfSe analysis, the relative abundances of Ruminococcaceae, *Oscillospira*, Rikenellaceae, Verrucomicrobia, Order YS2 [cyanobacteria], *Roseburia*, *Chryseobacterium*, Class 4C0d‐2 [cyanobacteria], Peptococcaceae and *Ruminococcus*were higher in SHS individuals than in the controls, indicating that bacteria could be potential biomarkers for SHS.

Several diseases can be diagnosed using intestinal microbiota. Faecal microbiota can be used to discriminate Parkinson's disease patients from controls with an AUC of 0.81 (95% CI: 0.72‐0.90), a sensitivity of 75.60% and a specificity of 77.80%.^61^ The intestinal microbiota was also used for the diagnosis of hypertension (AUC = 0.91, 95% CI: 0.75‐1.00)^21^ and early hepatocellular carcinoma (AUC = 0.81, 95% CI: 0.74‐0.87).^62^ Additionally, the microbiome was used for the diagnosis of several diseases, including CFS (AUC = 0.89)^28^ and ileal Crohn's disease (AUC = 0.97).^39^ In the present study, we also performed a supervised random forest tree (RF) model to diagnose SHS, resulting in an AUC (0.79, 95% CI: 0.77‐0.81). Recently, we demonstrated that plasma adrenaline/noradrenaline and cortisol can be used to diagnose SHS, with AUC values of 0.69 (95% CI: 0.65‐0.73) and 0.91 (95% CI: 0.89‐0.93), respectively.^63^ Therefore, the combination of intestinal microbiota and serum biochemicals, such as plasma adrenaline/noradrenaline and cortisol, might increase the capability of diagnosis using objective biomarkers.

The aetiology of SHS remains unclear; however, stress responses,^7^ which can trigger inflammation,^64^ are the primarily recognized mechanism. We previously demonstrated that SHS is associated with mRNA expression of glucocorticoid receptors in lymphocytes, which has been implicated in inflammatory responses.^11^ Increasing evidence has demonstrated that the disturbance of the intestinal microbiota can lead to systemic inflammation.^65^ In addition, different intestinal microbiota between the healthy and SHS individuals were identified in terms of the relative abundance of specific genera. Given these findings, abnormalities in intestinal microbiota might shape chronic inflammation in the gut, which subsequently leads to SHS. It highlights the association of specific taxa with SHS, and the identification of the underlying role of this altered commensal intestinal microbiota could supply novel diagnostic or therapeutic strategies.

There are several limitations in our study that may influence the results. First, in this study, we included more females than males, which might introduce selection bias in the association between intestinal microbiota and SHS. The prevalence of SHS is higher in females than that in males, and the response rate in faecal sample collection is also higher in females than in males. To increase the comparability between groups, we matched SHS cases and controls using propensity score matching based on age, gender and BMI. Second, the design of a case‐control study based on a cross‐sectional study makes it inevitable to avoid selection bias and may overestimate the accuracy of diagnosis for SHS. Third, the diet of participants, the main associated factor of the intestinal microbiota,^66^ was not controlled, which might introduce confounding bias. Forth, the cortisol and adrenaline/noradrenaline, the only known measurable biochemical parameter of SHS, were not included in our study. Finally, the sample size of our study is relatively small, resulting in the absence of statistical power. Therefore, further cohort studies with larger sample sizes are needed to explore the causal association between SHS and the intestinal microbiota.

## 5 CONCLUSION

In this case‐control study with a relatively small sample size, we first demonstrated that the intestinal microbiota is associated with SHS. Alteration of intestinal microbiota occurs with SHS, an early stage of disease, which might shed light on the importance of intestinal microbiota in the primary prevention of NCDs.

## CONFLICT OF INTEREST

The authors declare no conflicts of interest associated with this study.

## AUTHORS’ CONTRIBUTIONS

YW and WW contributed to the study design and concepts. QS, HL, XM, JL and JXZ contributed to the literature research. QS, HW, JZ, QL, HL and XX contributed to the data acquisition. QS, JZ, JL, QT, MS and QL contributed to the statistical analysis of data. QS, XZ, LW and WC contributed to the manuscript preparation. QS and YW contributed to the manuscript editing. All authors approved the manuscript.

## Data Availability

The data that support the findings of this study are available from the corresponding author upon reasonable request.
