# Supplementary figures and images for: Association of suboptimal health status with intestinal microbiota in Chinese youths
Source: J Cell Mol Med. 2019 Dec 6;24(2):1837–47. doi: 10.1111/jcmm.14880 (PMC6991644; doi:10.1111/jcmm.14880)

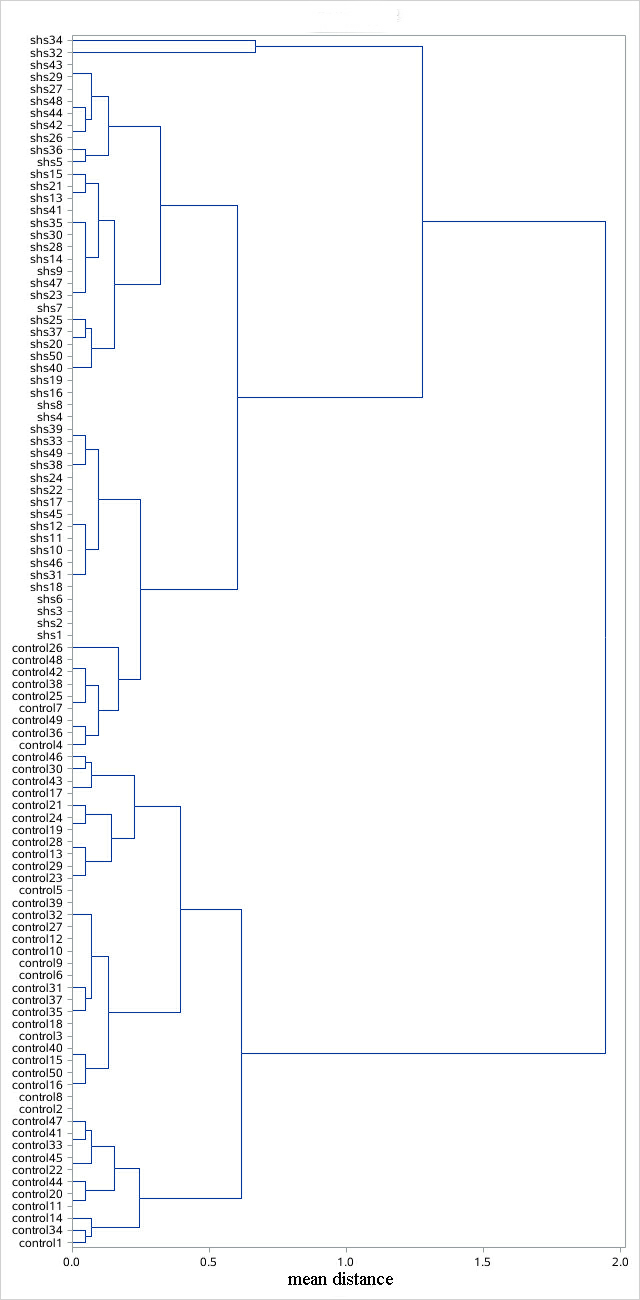

Supplement: Supplementary file 1 [file JCMM-24-1837-s001.tif]

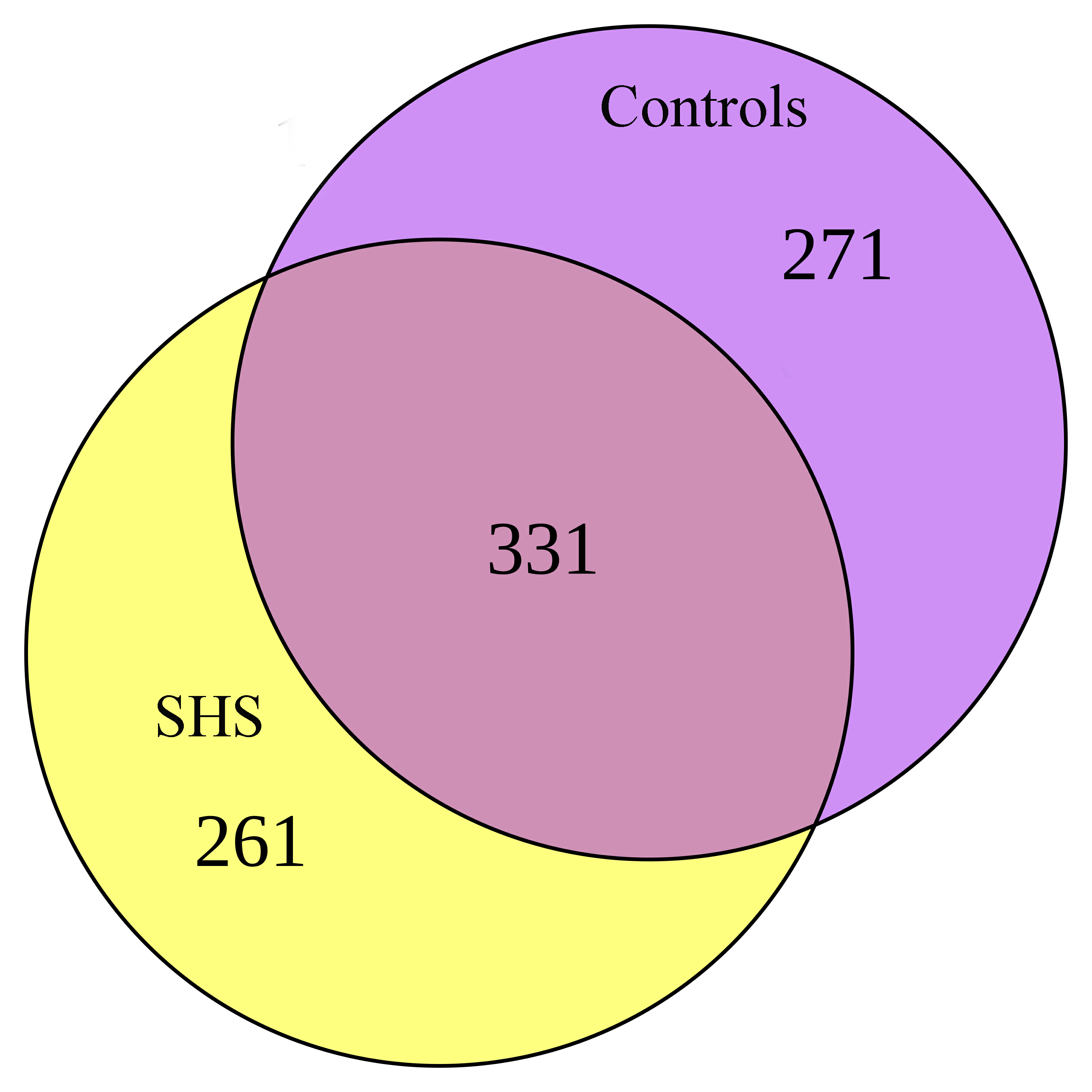

Supplement: Supplementary file 2 [file JCMM-24-1837-s002.tif]

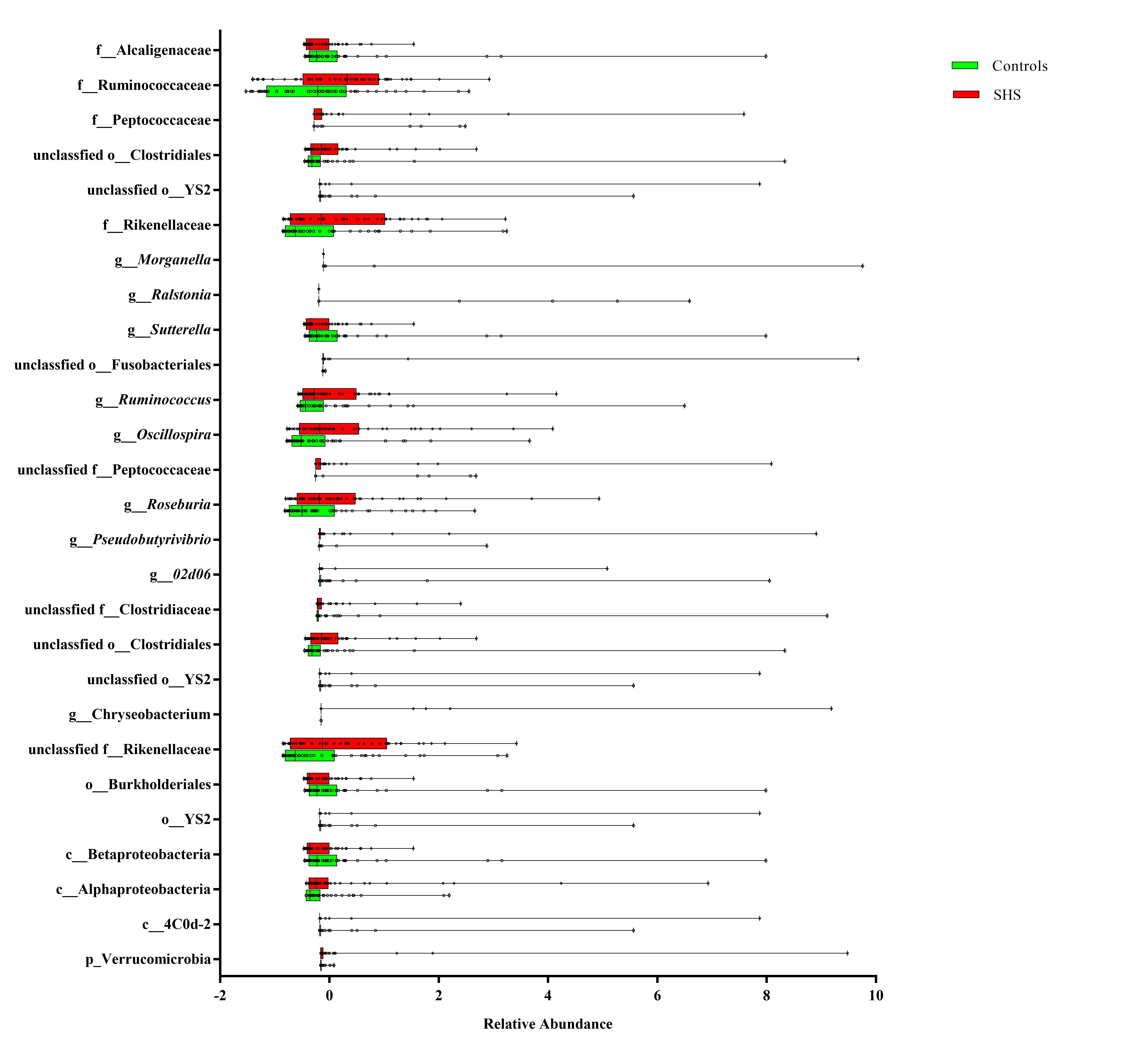

Supplement: Supplementary file 3 [file JCMM-24-1837-s003.tif]
